# Monolayer Fullerene Networks as Photocatalysts for Overall Water Splitting

**DOI:** 10.1021/jacs.2c08054

**Published:** 2022-10-19

**Authors:** Bo Peng

**Affiliations:** Theory of Condensed Matter Group, Cavendish Laboratory, University of Cambridge, J. J. Thomson Avenue, CambridgeCB3 0HE, United Kingdom

## Abstract

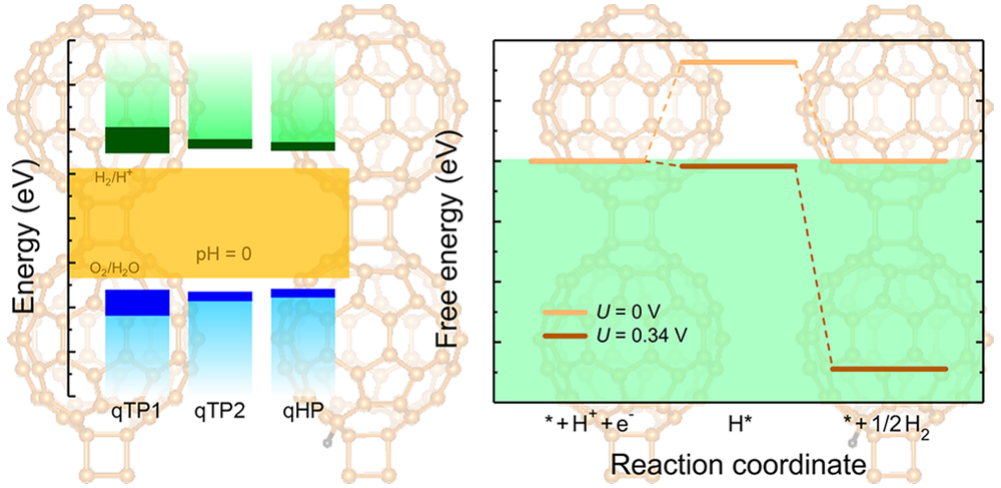

Photocatalytic water splitting can produce hydrogen in an environmentally friendly way and provide alternative energy sources to reduce global carbon emissions. Recently, monolayer fullerene networks have been successfully synthesized [Hou et al. *Nature***2022**, *606*, 507], offering new material candidates for photocatalysis because of their large surface area with abundant active sites, feasibility to be combined with other 2D materials to form heterojunctions, and the C_60_ cages for potential hydrogen storage. However, efficient photocatalysts need a combination of a suitable band gap and appropriate positions of the band edges with sufficient driving force for water splitting. In this study, I employ semilocal density functional theory and hybrid functional calculations to investigate the electronic structures of monolayer fullerene networks. I find that only the weakly screened hybrid functional, combined with time-dependent Hartree–Fock calculations to include the exciton binding energy, can reproduce the experimentally obtained optical band gap of monolayer C_60_. All the phases of monolayer fullerene networks have suitable band gaps with high carrier mobility and appropriate band edges to thermodynamically drive overall water splitting. In addition, the optical properties of monolayer C_60_ are studied, and different phases of fullerene networks exhibit distinct absorption and recombination behavior, providing unique advantages either as an electron acceptor or as an electron donor in photocatalysis.

## Introduction

The energy consumption of fossil fuels is the main source of global carbon emissions.^1^ As an alternative, hydrogen can be burnt in the presence of oxygen and produce only water, supporting mitigation of CO_2_ emissions. Photocatalysis can decompose water into hydrogen and oxygen using light, providing a low-cost approach for the green production of hydrogen. Photocatalytic water splitting has been extensively studied since the discovery of electrochemical photolysis of water in TiO_2_ in 1972.^2−11^ However, due to the wide band gap of 3.0–3.2 eV in TiO_2_, only the ultraviolet part of the solar spectrum can be harnessed. To maximize the photocatalytic efficiency, a water-splitting material needs to (*i*) absorb the light effectively to generate enough electron–hole pairs; (*ii*) separate the generated electrons and holes on the surface; and (*iii*) overcome the potential barrier of the reaction. For (*i*) and (*iii*), a compromise of the band gap is needed to harness the photon energy effectively while fulfilling the requirements of the band edges to facilitate the redox reaction of water. As a result, an optimal band gap around 2 eV is required, and the band edges must span the redox potential.^12−14^ For (*ii*), a type-II band alignment can spontaneously separate the electrons and holes. Based on these requirements, a variety of candidate materials have been proposed for efficient water splitting.^15−30^ Among all the candidates, carbon nanomaterials exhibit high physical stability and rich redox chemistry.^31,32^ In particular, fullerene, the cage structure of C_60_,^33^ displays high quantum efficiency in photocatalytic reactions because of their large surface area, abundant micropores, increased surface active sites, and efficient electron transport properties.^34−37^ In photocatalysis, C_60_ can enhance the photocatalytic activity via different mechanisms: it can work as an electron acceptor owing to rapid carrier separation,^36,38−40^ or as an energy transfer mediator,^41^ or as an electron donor due to high photosensitivity.^42^ In addition, for composite materials, the introduction of fullerene results in better crystallization by reducing the defects^37^ and can also improve the stability of the composites,^43,44^ which further enhance the photocatalytic efficiency. Most interestingly, C_60_ itself is a promising hydrogen storage material,^45−49^ and photocatalytic water splitting using fullerene provides a convenient approach to produce and store hydrogen at the same time.

Recently, a 2D material composed of covalently bonded fullerene network structures has been synthesized, with two configurations obtained: a few-layer quasi-tetragonal phase (qTP) and a monolayer quasi-hexagonal phase (qHP).^50^ The various structural phases of 2D fullerene networks can be combined with other 2D materials to form type-II van der Waals heterostructures,^51−53^ which can efficiently separate carriers between individual layers. In addition, the band alignment in these heterostructures can be further controlled by external strain because of the mechanical flexibility of 2D materials.^54−56^ Compared to heterostructures using C_60_ molecules where the low C_60_ content is not periodically bounded at the edge of the other 2D material,^57^ heterostructures using monolayer polymetric fullerene has a smooth microscopic surface with uniform periodic C_60_ networks, which provides higher crystallinity with higher C_60_ concentrations and consequently increases the photocatalytic activity. Compared to other 2D materials,^58−69^ monolayer C_60_ has larger surface area with more active sites due to the quasi-0D network structures of C_60_ cages. Additionally, monolayer C_60_ exhibits good thermodynamic stability and high carrier mobility.^50^ All these physical/chemical properties render monolayer fullerene networks a promising candidate for photocatalytic water splitting. However, all theoretical calculations underestimate the band gap of monolayer C_60_ by at least 10%,^54,55,70^ and a correct description of the band structures is the prerequisite for exploring the band edge positions for water splitting or the optical absorption for photocatalysis.

In this paper, the electronic structures of monolayer qTP and qHP fullerene networks are investigated using semilocal density functional theory (DFT) and hybrid functional calculations. By examining the band gap and exciton binding energy, I find that the electronic structures and optical properties of monolayer C_60_ can only be described correctly by a weakly screened hybrid functional. The band gaps of monolayer fullerene are around 1.67–1.88 eV, and the band edge positions of qTP C_60_ provide sufficient driving forces for overall water splitting. In addition, monolayer fullerene networks possess high carrier mobility that can effectively transfer the photoexcited electrons and holes. Furthermore, the carrier recombination in qTP C_60_ is suppressed by weak optical transitions, leading to efficient carrier separation as an electron acceptor. On the other hand, the strong optical absorption in qHP C_60_ can provide a large amount of electrons for hydrogen evolution, making it promising as an electron donor. These results indicate that monolayer fullerene networks are promising as efficient photocatalysts for overall water splitting.

## Methods

All crystal structures of monolayer fullerene networks are optimized using the PBEsol functional^71^ as implemented in vasp.^72,73^ A plane-wave cutoff of 800 eV is used with a **k**-mesh of 5 × 5 and 3 × 5 for qTP and qHP C_60_ respectively. During the structural relaxation, an energy convergence criterion of 10^–6^ eV and a force convergence criterion of 10^–2^ eV/Å are enforced. To mimic the 2D monolayers with 3D periodic boundary conditions, an interlayer vacuum spacing larger than 17 Å is used to eliminate interactions between adjacent unit cells along the *c* direction.

The electronic structures of qTP and qHP C_60_ are calculated using the screened hybrid functional HSE.^74−77^ Using the HSE wave functions, the partial (band decomposed) charge density is calculated for the top valence and bottom conduction bands at selected **k**-points. The transport properties are calculated based on the HSE eigenenergies and eigenstates in a **k**-mesh of 8 × 8 (5 × 8) for qTP (qHP) C_60_, which is further interpolated using an interpolation factor of 100. The scattering rates for acoustic deformation potential and ionized impurity scattering are calculated using the amset package.^78^ The deformation potential is calculated for the anisotropically contracted (−0.5%) and expanded (+0.5%) lattice, and the elastic tensor coefficients (including ionic relaxations) are computed using the finite differences method.^79,80^ For ionized impurity scattering, the static dielectric constant is calculated from density functional perturbation theory.^81^

When computing the optical properties, the thickness-independent absorbance *A*(ω) is calculated from the imaginary part of the dielectric function ϵ_2_(ω)^82−84^
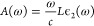
1where ω is the photon frequency, *c* is the speed of light, and *L* is the distance between the 2D sheets. The absorbance in the independent particle picture^81^ is calculated using the hybrid-functional electronic structures. To include the excitonic effects, time-dependent Hartree–Fock (TDHF) calculations are performed on top of the HSE eigenenergies and eigenstates using the Casida equation that includes couplings among the group of resonant/antiresonant two-orbital states.^85^ The exciton eigenenergies and their corresponding oscillator strengths can be obtained directly from the Casida equation.^85^ The exciton binding energy is then computed as the difference between the eigenenergy in the independent particle picture and the exciton eigenenergy. The Tamm–Dancoff approximation is used, as the exciton eigenenergies calculated within and beyond this approximation^86^ only have a difference smaller than 5 meV. In 2D materials, the exciton absorption spectrum calculated from TDHF agrees qualitatively well with the results obtained from the Bethe–Salpeter equation (BSE) on top of the *GW* calculations,^87^ and TDHF is computationally much less expensive than *GW* + BSE, especially for large systems such as monolayer fullerene networks. A **k**-mesh of 8 × 8 (5 × 8) is used for qTP (qHP) C_60_, with the highest eight (16) valence bands and the lowest eight (16) conduction bands included as the basis, converging the exciton eigenenergy within 1 meV.

To compute the thermodynamics of water adsorption and redox reactions, a supercell of 2 × 2 and 1 × 2 is used for qTP and qHP C_60_ respectively, with an electronic **k**-point grid of 3 × 3. Both the lattice constants and internal atomic coordination are fully relaxed for all the atoms. For hydrogen reduction reaction, geometry optimization always results in top-site adsorption. The lowest energy intermediates are evaluated by comparing hydrogen adsorption on all the symmetry irreducible carbon atoms. The thermal corrections at room temperature, including zero-point energy, entropy, and internal thermal energy, are calculated using vaspkit.^88^ The vibrational frequencies are computed for both the adsorbed hydrogen atoms and the neighboring carbon atoms within a radius of 2.5 Å.

## Results and Discussion

### Crystal Structures

**Figure 1:**
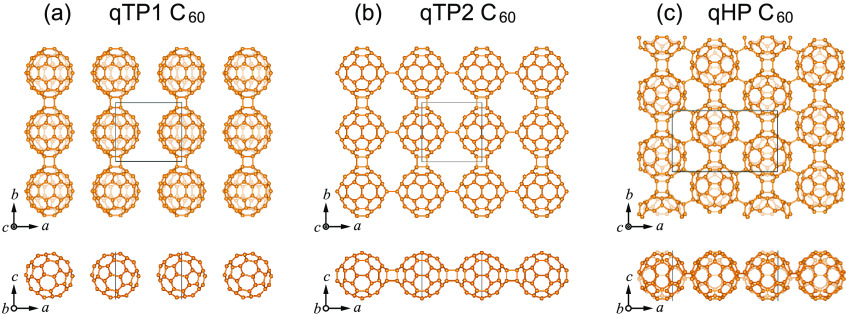
Crystal structures of monolayer (a) qTP1, (b) qTP2, and (c) qHP C_60_ from top and front views.

The crystal structures of fully relaxed fullerene networks are present in Figure 1. After geometry optimization, two quasi-tetragonal phases are obtained. One phase, denoted as qTP1, is obtained by structural relaxation starting from the quasi-tetragonal phase consisting of only carbon atoms. The other quasi-tetragonal phase, denoted as qTP2, is obtained by a two-step geometry optimization, which starts with the experimentally reported qTP Mg_2_C_60_ and then removes the Mg ions before the second relaxation. The two-step structural relaxation is to mimic the experimental procedure to remove the charged ions introduced during synthesis by treatment with hydrogen peroxide to obtain clean single crystals of the carbon polymers.^50,89^

Monolayer qTP1 C_60_ crystallizes in space group *P*2/*m* (No. 10) with lattice parameters *a* = 10.175 Å and *b* = 9.059 Å, in which each C_60_ is linked by two neighboring C_60_ cages through two [2 + 2] cycloaddition bonds along the *b* direction, forming 1D chains of C_60_ cluster cages in Figure 1a. The shortest interchain distance between the nearest carbon atoms is 3.065 Å along the *a* direction, which is much longer than the C–C single bonds. The interchain distance is shortened merely by 0.172 Å when including the van der Waals interactions;^90^ therefore, the van der Waal forces are neglected in qTP1 C_60_ (for the role of van der Waals forces in the lattice constants of all three phases, see the Supporting Information). The space group of qTP2 C_60_ is *Pmmm* (No. 47), with lattice parameters *a* = 9.097 Å and *b* = 9.001 Å. Similar to qTP1 C_60_, the in-plane [2 + 2] cycloaddition bonds connect neighboring C_60_ cages along the *b* direction in qTP2 C_60_. The difference between qTP1 and qTP2 C_60_ is along the *a* direction: no bond is formed between neighboring C_60_ chains in qTP1 fullerene along the *a* direction, whereas each C_60_ cage of qTP2 fullerene connects two neighboring cages along that direction through two out-of-plane [2 + 2] cycloaddition bonds, as demonstrated in Figure 1b. Monolayer qHP C_60_ has a space group of *Pc* (No. 7) with lattice parameters *a* = 15.848 Å and *b* = 9.131 Å, where each C_60_ is connected to six neighboring C_60_ cages with four C–C single bonds along the diagonal lines of the rectangular unit cell and two [2 + 2] cycloaddition bonds along the *b* direction, as demonstrated in Figure 1c. The calculated lattice constants agree well with previous calculations.^54^ The dynamic stability of all three phases is evaluated in the Supporting Information. In addition, the thermal stability of monolayer qTP and qHP C_60_ has been confirmed using molecular dynamics simulations in a previous study, showing that both qTP and qHP C_60_ monolayers can remain stable at temperatures near 800 K,^91^ which is in line with the experimental result that monolayer qHP C_60_ does not decompose at 600 K.^50^

### Appropriate Screening Parameter

To gain insight into the appropriate level of theory to correctly describe the electronic structures and optical properties of the C_60_ monolayers, the electronic and optical band gaps of monolayer qHP C_60_, as well as the exciton binding energy, are calculated using the hybrid functional with different screening parameters μ,^77,92−94^ and then compared with the experimentally determined value. In 2D materials, the excitonic effects are stronger than their bulk counterparts due to weaker dielectric screening^67,87,95^ (for dielectric screening in bulk and monolayer polymeric C_60_, see the Supporting Information). To include exciton binding energy, time-dependent Hartree–Fock calculations are performed on top of different hybrid functionals, which provides a qualitatively consistent exciton absorption spectrum compared to *GW* + BSE and is computationally much less expensive.^87^

**Figure 2:**
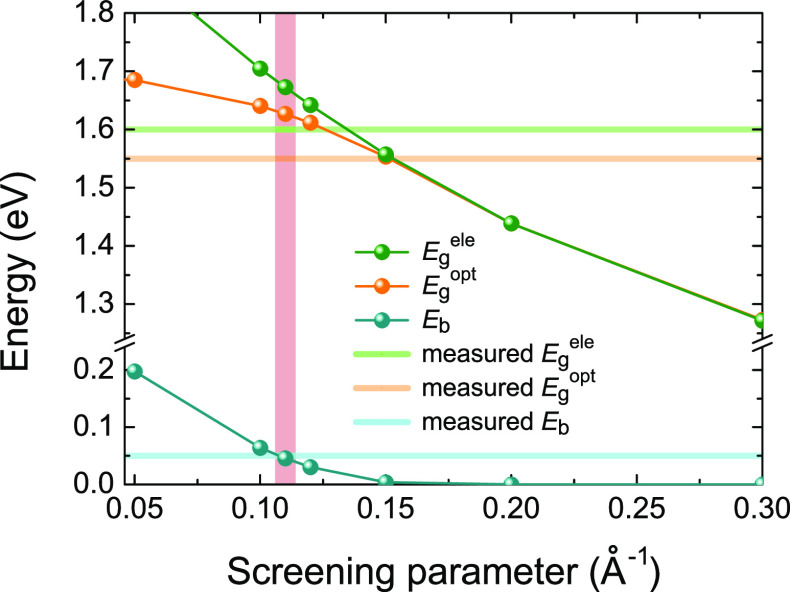
Electronic and optical band gaps, as well as the corresponding exciton binding energy, of monolayer qHP C_60_ calculated from different screening parameters.

Figure 2 summarizes the electronic band gap *E*_g_^ele^, optical band gap *E*_g_^opt^, and exciton binding energy *E*_b_ of qHP C_60_ computed from different screening parameters μ (for similar results on qTP C_60_, see the Supporting Information). A screening parameter larger than 0.15 Å^–1^ not only severely underestimates the electronic band gap *E*_g_^ele^ but also predicts zero exciton binding energy. For example, the HSEsol (the PBEsol counterpart of the widely used HSE06 with μ = 0.2 Å^–1^) hybrid functional predicts an electronic band gap of 1.44 eV, and the HSEsol band gap is 10% narrower compared to the measured gap of 1.6 eV, which can be attributed to an increase in the dielectric screening of HSEsol.^96^ Therefore, the HSEsol hybrid functional is inadequate to describe the electronic and optical properties of monolayer fullerene networks, as it tends to overestimate the screening effects in low-dimensional systems and consequently underestimate their band gap and exciton binding energy.^87,97,98^ This is unsurprising because in quasi-0D C_60_ monolayers the screening effects are much weaker than most 2D materials.

Among all the screening parameters below 0.15 Å^–1^, a screening parameter of 0.11 Å^–1^ yields an exciton binding energy *E*_b_ of 0.05 eV, which is in good agreement with the experimental value.^50^ The screening length is in excellent agreement with the inverse of the distance between two nearest neighboring buckyballs (∼9.1 Å). In addition, it predicts an electronic band gap of 1.67 eV compared to the measured *E*_g_^ele^ of 1.6 eV, while obtaining a reasonable *E*_g_^opt^ of 1.62 eV compared to the experimentally obtained 1.55 eV. The tiny discrepancy (<4.5%) may come from temperature effects such as electron–phonon coupling,^99−103^ which are not included in the calculations. Further decreasing the dielectric screening results in an overestimation of both the band gaps and the binding energy. Thus, a correct description of the band structures and optical properties can only be obtained by using the weakly screened hybrid functional with μ = 0.11 Å^–1^ and TDHF on top of the hybrid functional, respectively.

### Electronic Structures

**Figure 3:**
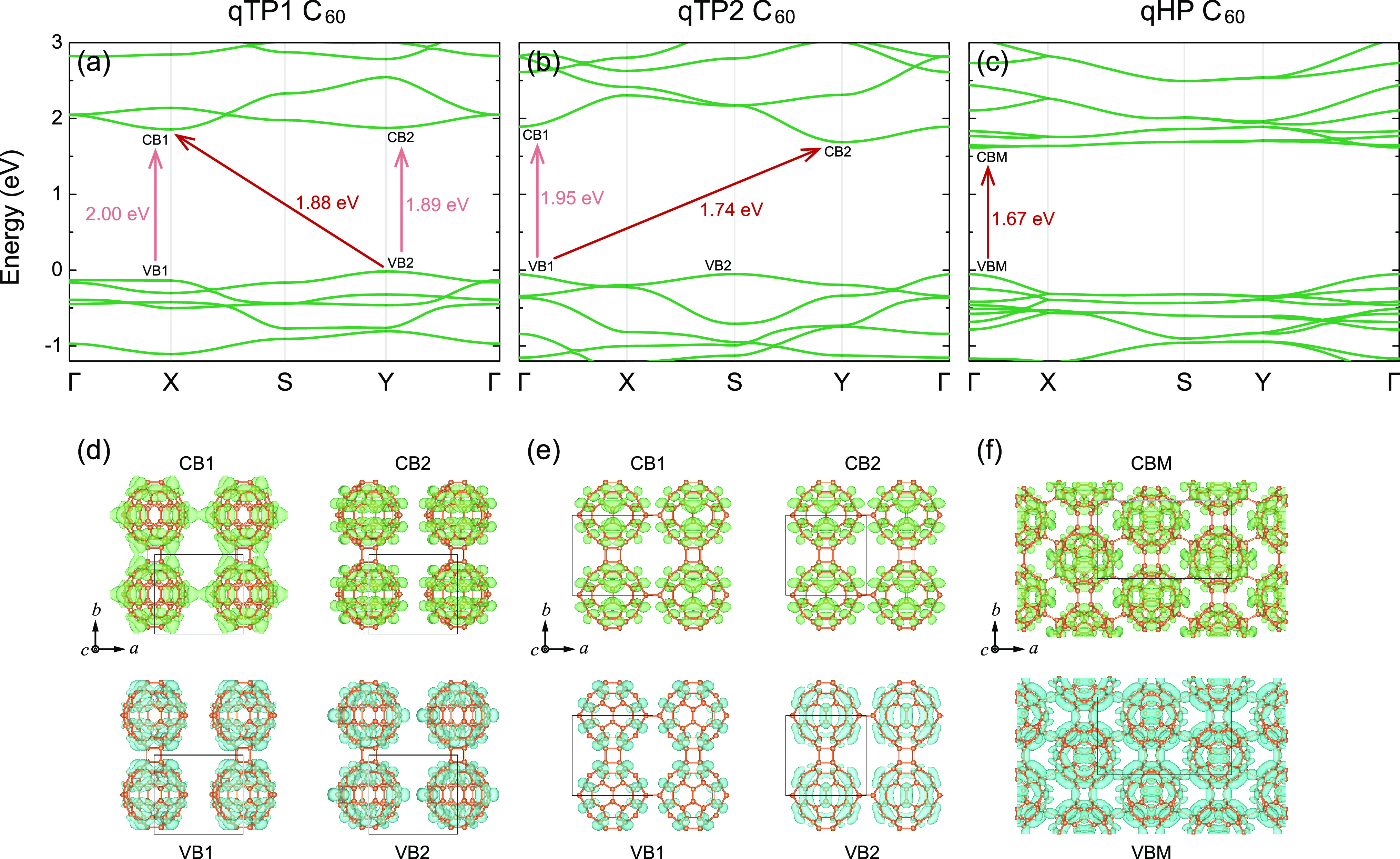
Electronic structures of (a) qTP1, (b) qTP2, and (c) qHP C_60_ calculated with weakly screened hybrid functional (μ = 0.11 Å^–1^), as well as their corresponding partial charge density of the top valence states and the lowest conduction states in (d)–(f). The default isosurface level is used (0.009 and 0.005 Å^–3^ for qTP and qHP C_60_ respectively), as implemented in vesta.^104^

Using the weakly screened hybrid functional with μ = 0.11 Å^–1^, the electronic structures are predicted (for band structures calculated from PBEsol and HSEsol, see the Supporting Information). All three phases have a 2D rectangular Brillouin zone (for details, see the Supporting Information), with high-symmetry points Γ (0, 0), X (1/2, 0), S (1/2, 1/2), and Y (0, 1/2). Figure 3a shows the band structures of qTP1 C_60_. The obtained band gap of 1.88 eV is indirect, with the valence band maximum (VBM) at the Y high-symmetry point and the conduction band minimum (CBM) at X. The direct transition energies at X and Y are 2.00 and 1.89 eV respectively.

To visualize the band edges, the partial charge density for the top valence and bottom conduction bands at X and Y is shown in Figure 3d. The lowest conduction band at X (CB1) is more dispersive, and its charge density is more diffuse along both the *a* and *b* directions. The highest valence band is flat along Γ – X, and as expected, the corresponding charge density of the top valence band at X (VB1) is isolated within separated C_60_ cages. Similarly, the top valence states and lowest conduction states at Y, denoted as VB2 and CB2 respectively, are centered around each single C_60_ cage, and such molecular-like character is consistent with their flat bands.

For qTP2 C_60_, the weakly screened hybrid functional predicts an indirect band gap of 1.74 eV with the VBM at Γ and the CBM at Y, while the direct transition energy at Γ is 1.95 eV. As shown in Figure 3b, the band structures of qTP2 C_60_ show distinct differences from qTP1 C_60_, despite the fact that their lattice parameters are similar. In addition, the charge density of qTP2 C_60_ changes significantly compared to that of qTP1 C_60_. Because the space group of qTP2 C_60_ (*Pmmm*) has more symmetry operations than that of qTP1 C_60_ (*P*2/*m*), their partial charge density in Figure 3e is more symmetric than that of qTP1 C_60_. Interestingly, although the lowest conduction band between Γ and Y has an energy difference of 0.21 eV, their corresponding partial charge density (denoted as CB1 and CB2 respectively) exhibits no significant difference. In contrast, for the highest valence band, although the energy difference between Γ and S is lower than 0.7 meV, their partial charge density (denoted as VB1 and VB2 respectively) is distinct from each other.

Figure 3c depicts the band structures of monolayer qHP C_60_. Monolayer qHP C_60_ possesses a direct band gap at Γ. The CBM of monolayer qHP C_60_ exhibits flat-band features, and its charge density is molecular-like, as shown in Figure 3f. On the other hand, the charge of the more dispersive VBM is distributed in the entire Brillouin zone, connecting neighboring C_60_ cages via both the C–C single bonds and the [2 + 2] cycloaddition bonds. Therefore, holes are expected to diffuse more effectively in qHP C_60_.

### Carrier Mobilities

**Figure 4:**
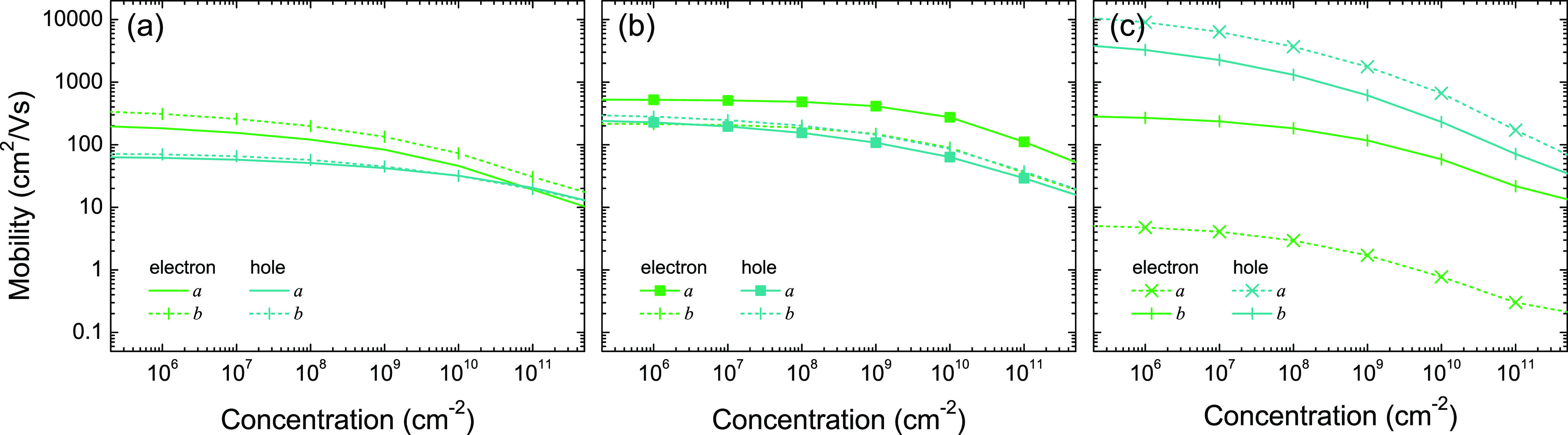
Mobility of monolayer (a) qTP1, (b) qTP2, and (c) qHP C_60_ at 300 K as a function of carrier concentration.

To confirm the transport properties, the carrier mobilities of all three phases at 300 K are calculated as a function of carrier concentration. As shown in Figure 4, the mobilities for both electrons and holes decrease with increasing carrier concentration in all three phases, as ionized impurity scattering becomes stronger. Although the experimental carrier concentration is unknown, the calculated electron mobility along *a*, 1.7–4.8 cm^2^/(V s) at low carrier concentrations (<10^9^ cm^–2^), is in perfect agreement with the measured electron mobility.^50^

The obtained electron mobility for qTP1 C_60_ in Figure 4a is higher than the hole mobility in a wide doping range, consistent with the more dispersive CB1 in Figure 3a. For both electrons and holes, the mobility along the 1D chain (*b* direction) is higher than that perpendicular to the chain (*a* direction). For qTP2 C_60_ in Figure 4b, the electron mobility along *a* is the highest. This is unsuprising because the CB1 along Γ–X and CB2 along S–Y are more dispersive than other bands in Figure 3b and both states along *a* in Figure 3e tend to overlap across the vertical [2 + 2] cycloaddition bonds. For qHP C_60_, the hole mobilities along both directions are much higher than the electron mobilities, as shown in Figure 4c, which is in line with the dispersive VBM at Γ along both directions in Figure 3c and the corresponding diffuse charge density in Figure 3f. The electron mobility along *a* is much lower than that along *b* because the CBM along Γ–X is much flatter than that along Y−Γ. Despite that, even the lower bound of the mobility is still relatively high, as the nonlocalized π bonds in C_60_ allow efficient carrier transfer.^31^

### Optical Absorption

**Figure 5:**
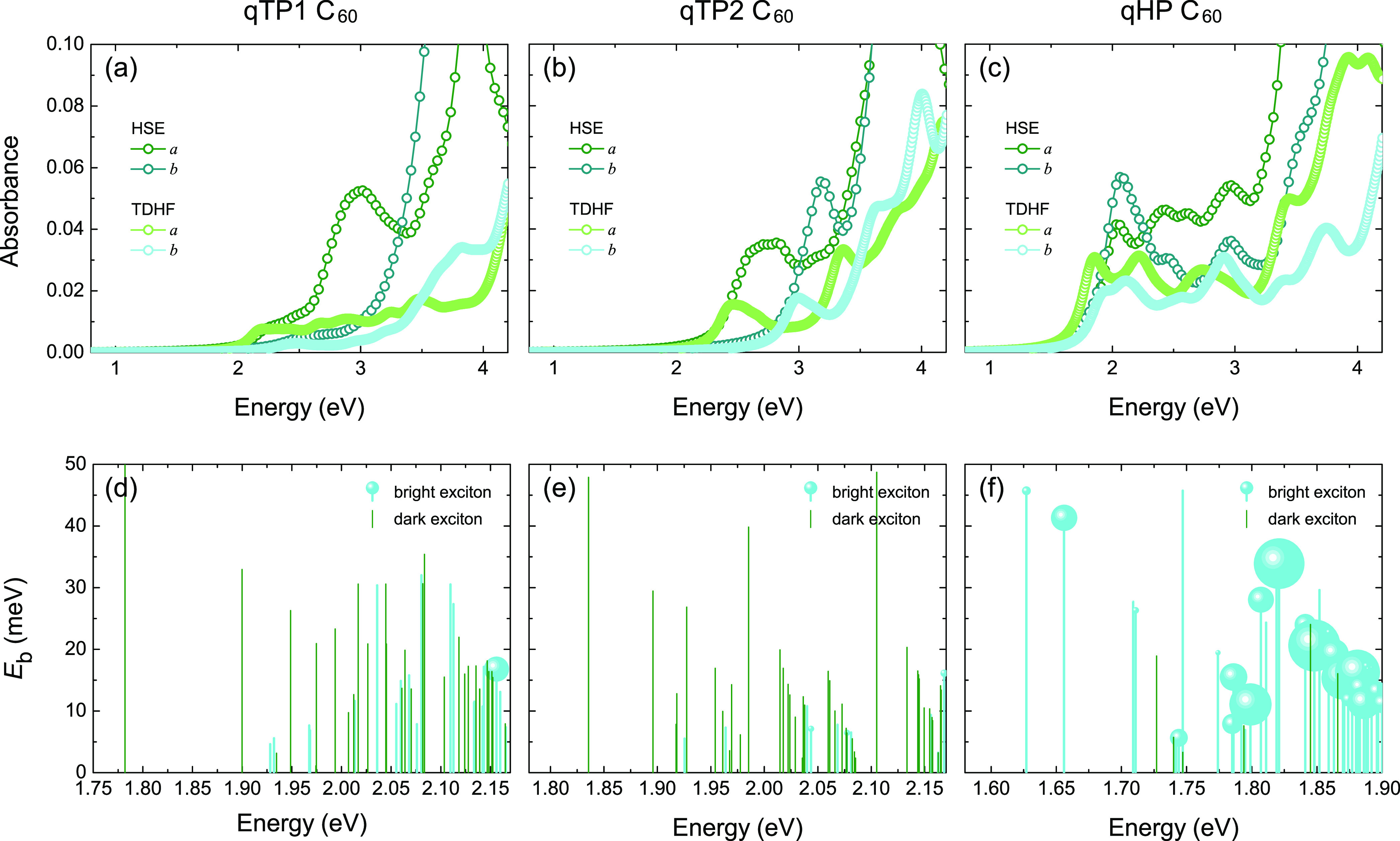
Absorbance of monolayer (a) qTP1, (b) qTP2, and (c) qHP C_60_ calculated with HSE (μ = 0.11 Å^–1^) and TDHF on top of HSE, as well as the binding energy *E*_b_ of the low-energy excitons in (d)–(f). The radius of the bright excitons indicates the oscillator strength. The larger the radius, the higher the oscillator strength.

Having established that all three fullerene networks can separate the carriers effectively in 2D, their absorption spectra for photocatalysis are then investigated. The thickness-independent absorbance *A*(ω) of monolayer fullerene networks is first calculated by using the weakly screened hybrid functional with μ = 0.11 Å^–1^, corresponding to the optical absorption of the hybrid-functional electronic structures in the independent particle picture. The absorbance of all three phases is gathered in Figure 5a–c. Within the independent particle approximation, the low-energy absorbance of both qTP1 and qTP2 C_60_ is strongly anisotropic along the *a* and *b* directions, whereas the first absorbance peaks of qHP C_60_ have similar energies along both directions. Moreover, the indirect band gaps of qTP1 and qTP2 C_60_, along with the low optical transition probabilities between the highest valence and lowest conduction bands, give rise to low optical absorbance below 2 eV.

Beyond the independent particle approximation, the absorbance is evaluated by HSE + TDHF to assess the excitonic contributions, as demonstrated by the green and cyan curves in Figure 5a–c. In monolayer qTP1 C_60_, the inclusion of excitonic effects leads to a much weaker optical absorbance, as shown in Figure 5a. This is because the low-energy excitons are mostly dark and the optical transitions involved in these dark excitons have zero oscillator strengths, as demonstrated in Figure 5d. For monolayer qTP2 C_60_, although the oscillator strengths in the low-energy range are mostly zero in Figure 5e, the exciton absorbance peak in monolayer qTP2 C_60_ is only moderately weaker than the independent particle absorbance peak in Figure 5b. Compared to its qTP counterparts, much stronger exciton absorbance peaks are observed in monolayer qHP C_60_, as shown in Figure 5c. The low-energy excitons in monolayer qHP C_60_ are mostly bright with binding energies around 5–50 meV, as present in Figure 5f. Therefore, strong exciton absorbance is induced in qHP C_60_, and in particular, the absorbance around 2 eV (0.20–0.32) is even stronger than those in zero band gap graphene^82,105^ and in large band gap photocatalysts such as monolayer GaSe^106^ and blue phosphorus/Mg(OH)_2_ van der Waals heterostructures,^107^ which makes qHP C_60_ a promising photocatalytic material to effectively utilize the solar spectrum around 2 eV.

### Band Alignment

**Figure 6:**
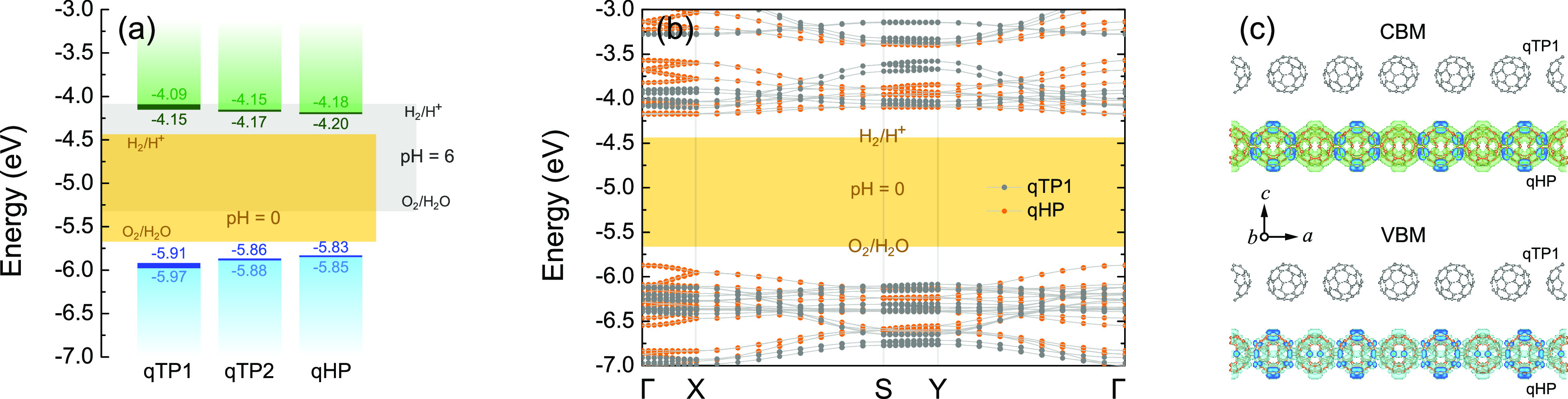
(a) Band alignment of qTP1, qTP2, and qHP C_60_ monolayers calculated with HSE (μ = 0.11 Å^–1^). The CBM and VBM positions in the independent particle picture are marked in green and cyan respectively, while the CBM and VBM positions including the excitonic effects are marked in dark green and blue, respectively. (b) Band structures of qTP1/qHP heterostructures, with the vacuum level set to zero. (c) Partial charge density of the CBM and VBM states in the qTP1/qHP heterostructures. The default isosurface level (0.002 Å^–3^) is used, as implemented in vesta.^104^

The exciton absorbance peaks in monolayer qHP C_60_ networks around 2 eV can maximize the solar energy absorption for water splitting.^12,14^ For an overall water splitting reaction, the energy levels of the CBM and VBM must straddle the redox potentials of water. In other words, the CBM (with respect to the vacuum level) should be higher than the hydrogen evolution potential of −4.44 + pH × 0.059 eV, while the VBM should be lower than the oxygen evolution potential of −5.67 + pH × 0.059 eV.^65,69,108^ To determine the band edge positions of qTP1, qTP2, and qHP C_60_ monolayers, the vacuum levels of all three phases are calculated by averaging the electrostatic potential along the *c* axis. Figure 6a summarizes the HSE band alignment of all three C_60_ monolayers with μ = 0.11 Å^–1^ (for band alignment calculated with PBEsol, HSEsol and unscreened hybrid functional, see the Supporting Information). In monolayer qTP1 C_60_, the CBM is 0.35 eV higher than the reduction reaction potential of H_2_/H^+^ at pH = 0, which is suitable for water reduction. Moreover, the VBM is 0.30 eV lower than the oxidation potential of O_2_/H_2_O at pH = 0, which is suitable for water oxidation. Similarly, the CBM of qTP2 C_60_ is 0.29 eV higher than the reduction potential and the VBM is 0.22 eV lower than the oxidation potential. Regarding monolayer qHP C_60_, the CBM lies 0.26 eV above the reduction potential and the VBM is 0.18 eV below the oxidation potential. Including the exciton binding energy leads to band edge shifts toward the redox potential by 0.06 eV for qTP1 C_60_, while the band edge shifts in qTP2 and qHP C_60_ are about 0.02 eV. Therefore, all three C_60_ monolayers exhibit large band gaps with appropriate band edge positions for overall photocatalytic water splitting at pH = 0. Increasing the pH upshifts the redox potentials of water, and at pH = 6, all three phases of monolayer C_60_ are no longer suitable for water reduction.

The lattice parameters of 3 × 1 qTP1 C_60_ and 2 × 1 qHP C_60_ are matched within 3.8% for *a* and 0.8% for *b* respectively. Therefore, monolayer qTP1 and qHP C_60_ can be combined to form qTP1/qHP heterostructures. To identify the type of the heterostructures for device applications, the band alignment at the qTP1/qHP interface is investigated. Compared to qTP1 C_60_, qHP C_60_ has a consistently smaller band gap, as shown in Figure 6a. The offset between the conduction band edges of qTP1 and qHP C_60_ monolayers is 0.09 eV with the CBM of qHP lower than that of qTP1, and a higher VBM of qHP relative to qTP1 leads to a valence band discontinuity of 0.12 eV. Consequently, a type-I (straddling gap) band alignment exists between qTP1 and qHP C_60_. Geometry optimization of the qTP1/qHP heterostructures results in 3.5% strain along *a* and 0.3% strain along *b* for qTP1 C_60_, while compresses the qHP C_60_ lattice by 0.4% and 0.5% along *a* and *b* respectively (for strain effects on band alignment of individual monolayers, see the Supporting Information). Despite that, the band alignment is still type-I, as demonstrated in Figure 6b. The type-I heterostructures with qTP1 and qHP C_60_ can be utilized in optical devices such as light-emitting diodes owing to high emission efficiency,^109^ or in lasers because of efficient recombination of spatially confined electrons and holes.^51^ As confirmed by the partial charge density of CBM and VBM in Figure 6c, these states are confined in monolayer qHP C_60_.

### Thermodynamic Driving Force for Water Splitting

The thermodynamics of water adsorption on monolayer fullerene networks are investigated by calculating the total energy difference between the H_2_O-adsorbed C_60_ and individual systems (i.e., pristine monolayer C_60_ and isolated H_2_O molecule).^26^ The obtained adsorption energies for qTP1, qTP2, and qHP C_60_ are −0.151, −0.109, and −0.107 eV respectively, indicating their capability of water adsorption.

**Figure 7:**
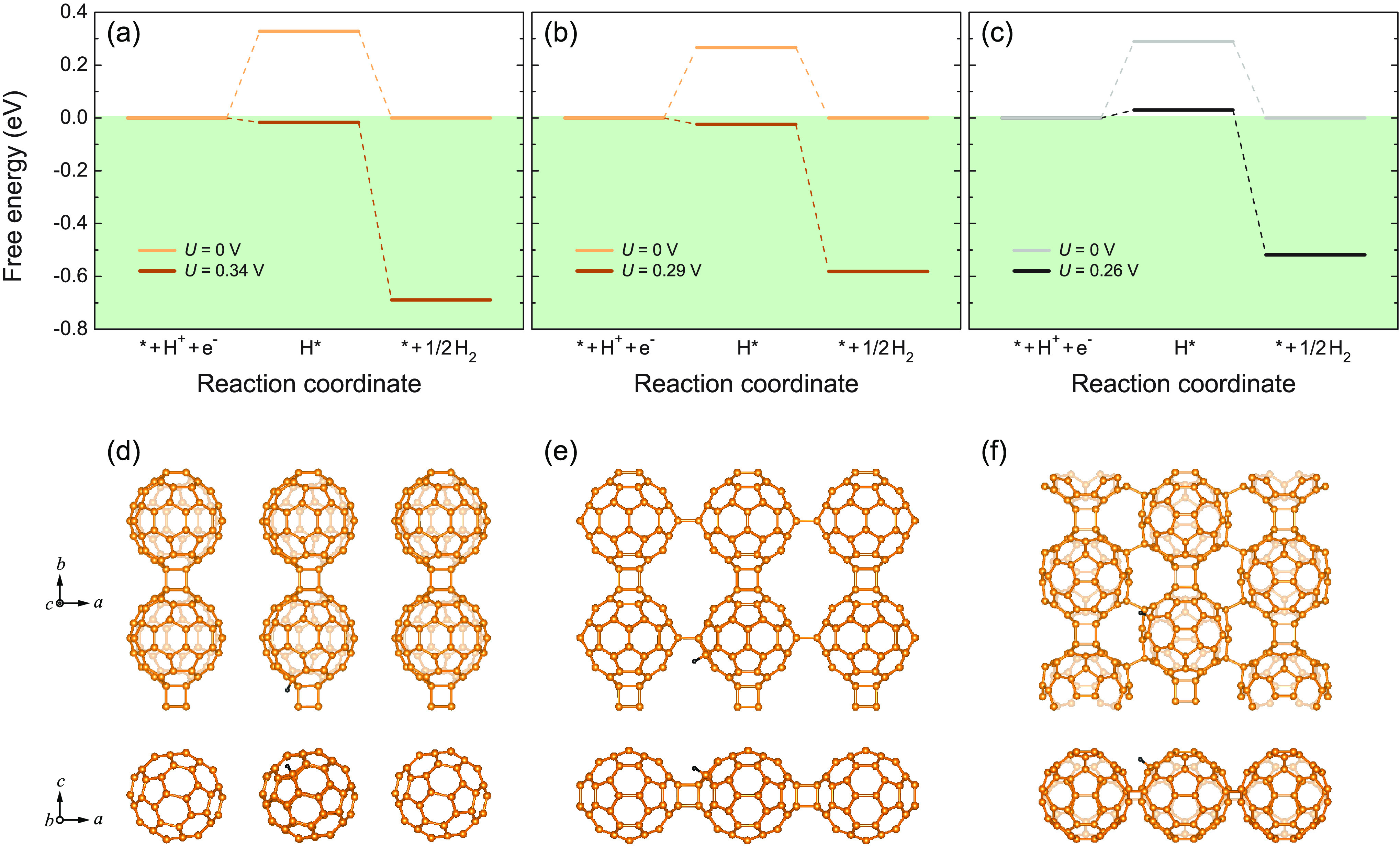
Free-energy diagram for hydrogen reduction reaction at pH = 0 and room temperature in (a) qTP1, (b) qTP2, and (c) qHP C_60_, with the Gibbs free energy of the combination of monolayer fullerene networks, a proton and an electron set to zero. *U* = 0 V corresponds to the absence of photoexcitaion. The nonzero potential *U* is generated by photoexcited electrons in the CBM. The lowest energy intermediates H* for all three phases are present in (d)–(f).

The thermodynamics of the hydrogen evolution reaction are investigated by calculating the Gibbs free energy of the intermediates of the reaction at pH = 0 and room temperature^15,16^ (for details on the half-reaction of water oxidation, see the Supporting Information). As shown in Figure 7a–c, the hydrogen evolution reaction has two steps. In the first step, monolayer fullerene networks (denoted as *) combine with a proton (H^+^) and an electron (e^–^) to form H* species. In the second step, H_2_ molecules are formed from the H* species. The lowest energy intermediates H* for all three phases are present in Figure 7d–f. For qTP1 C_60_, the hydrogen atom is adsorbed at the top site of the nearest neighboring carbon atom to the [2 + 2] cycloaddition bonds. Similarly, the adsorbed H atom on qTP2 C_60_ is at the top site of the nearest neighboring carbon atom to the vertical [2 + 2] cycloaddition bonds. Different from qTP1 and qTP2 C_60_, in the H-adsorbed qHP C_60_, a C–H bond is formed between the hydrogen atom and the second nearest neighboring carbon atom to the C–C single bond.

In the absence of photoexcitation (*U* = 0 V), all three phases of monolayer fullerene networks, when forming the lowest energy H* species, exhibit unfavorable positive Gibbs free energies (0.327, 0.266, and 0.289 eV for qTP1, qTP2 and qHP C_60_ respectively). Then the release of H_2_ molecules from the H* species is exothermic. Upon light irradiation, the photoexcited electrons in the CBM generate an external potential *U* of 0.345, 0.291, and 0.259 eV for qTP1, qTP2, and qHP C_60_ respectively, corresponding to the potential difference between the CBM and the H_2_/H^+^ reduction potential. Consequently, both steps (the formation of H* species and the release of H_2_ molecules) in the hydrogen reduction reaction in the free-energy diagram are downhill for qTP1 and qTP2 C_60_. Therefore, both qTP1 and qTP2 C_60_ can efficiently split water under an acidic environment upon light irradiation as the hydrogen reduction reaction can spontaneously proceed. Regarding qHP C_60_, the reaction barrier is significantly reduced to 0.030 eV under photoexcitation, which is close to the thermal fluctuation energy *k*_B_*T* at room temperature (0.026 eV). In addition, it has been reported that the experimentally obtained qHP C_60_ flakes tend to be negatively charged,^50,89^ which can provide further external potential for hydrogen evolution reaction.

### Discussion

Monolayer fullerene networks can be combined with a highly diverse set of lattice-matched 2D materials with higher CBM and VBM^51−53,59^ to form type-II heterostructures to separate electrons and holes in individual layers, which can further improve the photocatalytic performance (for type-II band alignment of qTP2/SnTe and qTP2/PbTe heterostructures, see the Supporting Information). The presence of monolayer fullerene networks can improve the separation of electrons and holes by trapping them individually into different nanostructures, i.e. 0D C_60_ cages in all three phases, or 1D C_60_ chains in qTP1 fullerene. For the 0D C_60_ cages in all three phases, the nonlocalized π bonds in C_60_ allow continuous transfer and separation of the photogenerated carriers.^31^ Furthermore, the enhanced surface area in monolayer fullerene networks, with more micropores and surface active sites compared to other 2D materials, can significantly increase the photocatalytic efficiency. Additionally, the optical transition oscillator strength in both the qTP1 and qTP2 monolayers is quite low, thereby suppressing the carrier recombination and enhancing the photocatalytic efficiency as an electron acceptor. Regarding monolayer qHP C_60_, the strong optical absorbance can generate a large amount of electrons, making it promising for providing electrons for hydrogen evolution.

Most interestingly, fullerene itself, after doping^45,49^ or coating,^46,47^ can act as promising molecular hydrogen attractors. Theoretical calculations have reported that one transition metal atom bound to fullerene can bind 11 hydrogen atoms, with a binding energy of 0.3 eV that is ideal for vehicular applications because of its ability to adsorb and desorb H_2_ reversibly.^45^ In addition, the maximum hydrogen storage density can reach 6–9 wt % near ambient pressure at room temperature,^45−47^ which is highly desirable for fuel-cell powered vehicles. Moreover, there is both theoretical and experimental evidence that fullerene can be decorated with various metal atoms while remaining stable.^110,111^ In monolayer fullerene networks, the decorating atoms can be uniformly distributed to form monolayer coating, which may further increase the retrievable hydrogen storage density.

## Conclusion

In summary, a weakly screened hybrid functional is used to examine the band structures of monolayer C_60_, rationalizing the measured electronic band gap. On top of the hybrid-functional electronic structures, time-dependent Hartree–Fock calculations predict excellent exciton binding energy, reproducing the measured optical band gap. To gain insights into the photocatalytic performance of monolayer fullerene networks, I investigate the band alignment of monolayer fullerene networks, and find that all three phases have the band edge positions suitable for overall water splitting. The overall water splitting can occur spontaneously in qTP C_60_ under acidic conditions at room temperature upon photoexcitation. The distinct optical properties between qTP and qHP fullerene provide unique advantages for different applications in photocatalysis, with qTP C_60_ being a likely electron acceptor and qHP C_60_ being a promising electron donor, respectively. Beyond water splitting, the type-I band alignment for the qTP1/qHP heterostructures offers new opportunities for optical devices and lasers.

## Supporting Information Available

A. Lattice constants with and without van der Waals interactions. B. Brillouin zone for qTP1, qTP2 and qHP C_60_. C. Dynamic stability. D. Screening parameters for qTP C_60_. E. Dielectric screening in polymeric C_60_. F. Band structures of 2D C_60_ calculated from PBEsol, HSEsol, and PBEsol0. G. Band alignment calculated with PBEsol, HSEsol, and PBEsol0. H. Half-reaction of water oxidation in qHP C_60_. I. Type-II band alignment of qTP2/SnTe and qTP2/PbTe heterostructures. (PDF)
