# Supplementary material for: Monolayer Fullerene Networks as Photocatalysts for Overall Water Splitting
Source: J Am Chem Soc. 2022 Oct 19;144(43):19921–31. doi: 10.1021/jacs.2c08054 (PMC9634807; doi:10.1021/jacs.2c08054)
Supplement: Supplementary file 1 — ja2c08054_si_001.pdf [file ja2c08054_si_001.pdf]

Supporting Information for "Monolayer fullerene networks as photocatalysts for overall water splitting"

Bo Peng[1,*]

[1] *Theory of Condensed Matter Group, Cavendish Laboratory, University of Cambridge, J. J. Thomson Avenue, Cambridge CB3 0HE, United Kingdom*

(Dated: October 9, 2022)

CONTENTS

A. Lattice constants with and without van der Waals interactions 2

B. Brillouin zone for qTP1, qTP2 and qHP C_{60} 3

C. Dynamic stability 3

D. Screening parameters for qTP C_{60} 4

E. Dielectric screening in polymeric C_{60} 5

F. Band structures of 2D C_{60} calculated from PBEsol, HSEsol and PBEsol0 5

G. Band alignment calculated with PBEsol, HSEsol and PBEsol0 7

H. Half-reaction of water oxidation in qHP C_{60} 8

I. Type-II band alignment of qTP2/SnTe and qTP2/PbTe heterostructures 8

References 9

A. Lattice constants with and without van der Waals interactions

To understand the role of van der Waals interactions in the computed crystal structures, the lattice constants are calculated using the zero damping DFT-D3 method of Grimme [1]. As shown in Table S1, the van der Waals forces have negligible effects on the lattice constants. Apart from the interchain lattice constant a of qTP1 C_{60} that is decreased by 0.172 Å (1.7%), all the other lattice constants are decreased by less than 0.3% after including the van der Waals forces in the geometry optimization. For qTP C_{60}, the lattice constants obtained without including the van der Waals interactions agree better with the measured distance between adjacent C_{60} centres of 9.1 Å [2].

TABLE S1. Calculated lattice constants of qTP1, qTP2 and qHP C_{60} monolayers with and without van der Waals interactions.

	a PBEsol	b PBEsol	a PBEsol-D3	b PBEsol-D3
qTP1	10.175	9.059	10.003	9.045
qTP2	9.097	9.001	9.080	8.979
qHP	15.848	9.131	15.798	9.110

B. Brillouin zone for qTP1, qTP2 and qHP C_{60}

As shown in Fig. S1, all three phases have a 2D rectangular Brillouin zone with high-symmetry points Γ (0, 0), X (1/2, 0), S (1/2, 1/2) and Y (0, 1/2).

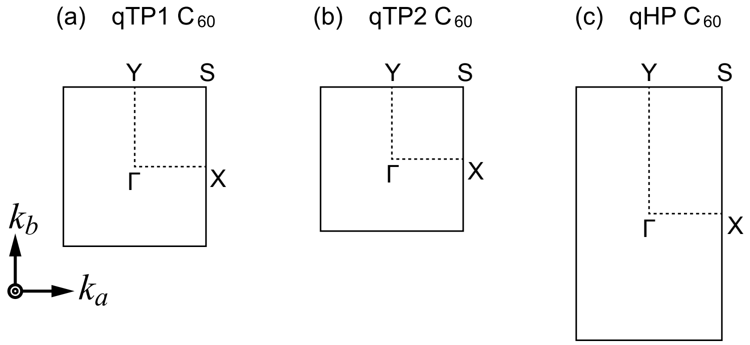

FIG. S1. 2D rectangular Brillouin zone of (a) qTP1, (b) qTP2, and (c) qHP C_{60}.

C. Dynamic stability

The force constants are calculated from density functional perturbation theory[3–5]. A 2×2 supercell with an electronic **k**-point grid of (Γ-centered) 2×2 is used for qTP C_{60}, while a 1×2 supercell is used for qHP C_{60}. The phonons are computed from the force constants under the harmonic approximation using the PHONOPY code[6,7].

The dynamic stability of all three phases is evaluated in Fig. S2. For monolayer qTP1 C_{60}, there is a fourth mode with almost zero frequency (0.30 THz) at Γ, corresponding to the rotation of 1D chains. Such nearly zero-frequency mode indicates the quasi-1D nature of qTP1 C_{60} similar to the torsional acoustic mode in 1D materials[8,9]. The torsional mode becomes slightly imaginary ($< 0.3i$ THz) along the Γ-X high-symmetry line, corresponding to the individual rotation of each 1D chain because of the lack of interchain bonding along a. Therefore, no free-standing qTP1 C_{60} monolayer has been reported experimentally[2], and such phase might only exist on the substrates. On the other hand, both qTP2 and qHP C_{60} exhibit no imaginary mode, confirming their dynamic stability.

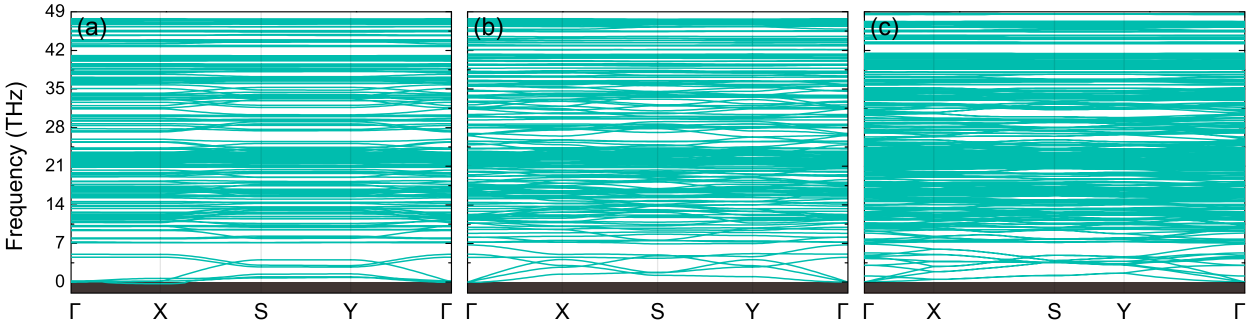

FIG. S2. Phonon spectra of monolayer (a) qTP1, (b) qTP2 and (c) qHP C_{60}.

D. Screening parameters for qTP C_{60}

Figure S3 summarizes the electronic band gap E_g^{ele}, optical band gap E_g^{opt} and exciton binding energy E_b of qTP C_{60} computed from different screening parameters μ. Similar to the case in qHP C_{60}, a screening parameter larger than $\mu = 0.2$ Å^{-1} leads to zero exciton binding energy. Therefore, $\mu = 0.2$ Å^{-1} (the PBEsol counterpart of the widely used HSE06) or $\mu = 0.3$ Å^{-1} (the PBEsol counterpart of the HSE03) is inadequate to describe the electronic and optical properties of monolayer fullerene networks. In the main text, the discussion of the electronic structures, optical absorption and band alignment is based on the weakly screened hybrid functional with $\mu = 0.11$ Å^{-1}.

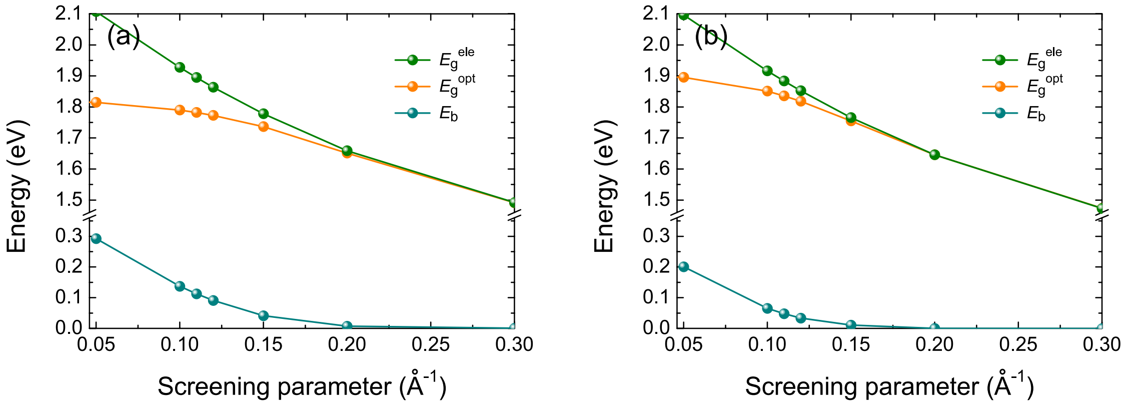

FIG. S3. Electronic and optical band gaps of monolayer (a) qTP1 and (b) qTP2 C_{60} calculated from different screening parameters, as well as the corresponding exciton binding energy.

Because of the quasi-1D nature of qTP1 C_{60}, its exciton binding energy (0.11 eV) is much higher than qTP2 C_{60} (0.05 eV). This is because the dielectric screening in quasi-1D qTP1 C_{60} is weaker than the tightly-bound qTP2 C_{60} networks. In the next section, the dielectric screening of all three phases is investigated, along with their bulk counterparts.

E. Dielectric screening in polymeric C_{60}

The static dielectric tensor is calculated from density functional perturbation theory [10]. For qTP and qHP C_{60} monolayers, a nominal layer thickness of 14.78 Å and 16.94 Å is used respectively, corresponding to the interlayer distance of their bulk counterparts. As listed in Table S2, the static dielectric constants in 2D polymeric C_{60} are much lower than their bulk counterparts. This is consistent with the fact that the weakly screened hybrid functional with $\mu = 0.11$ Å^{-1} provides the correct description of the electronic structures and optical properties, while the HSEsol overestimates the screening effects and leads to much smaller band gaps and zero exciton binding energy.

TABLE S2. Calculated static dielectric constants of 2D qTP1, qTP2 and qHP C_{60} monolayers and their bulk counterparts.

	2D qTP1	2D qTP2	2D qHP	3D qTP	3D qHP
ϵ_{xx}	3.01	3.91	4.76	6.38	11.42
ϵ_{yy}	3.60	3.80	4.40	7.36	7.33
ϵ_{zz}	-	-	-	6.06	67.83

F. Band structures of 2D C_{60} calculated from PBEsol, HSEsol and PBEsol0

The electronic structures of qTP and qHP C_{60} are calculated using the PBEsol functional [11] and the screened hybrid functional HSEsol [12]. The band structures of qTP1 C_{60} are shown in Fig. S4(a). The obtained PBEsol and HSEsol band gaps are 1.09 and 1.65 eV respectively, with the valence band maximum (VBM) at the Y high-symmetry point and the conduction band minimum (CBM) at X. The obtained PBEsol partial charge density of the top valence states and the lowest conduction states in Fig. S4(d) shows no distinct difference from the HSE partial charge density in the main text. The lowest conduction band at X (CB1) is more dispersive compared to other bands (VB1, VB2 and CB2), and the charge density of CB1 is more diffuse along both the a and b directions.

As both PBEsol and HSEsol functionals tend to overestimate the screening effects in low-dimensional systems and consequently underestimate their band gap [13–15], the band gap is also evaluated by using unscreened hybrid functional PBEsol0, in which the Hartree-Fock and PBEsol exchange energies are mixed in a 1:3 ratio along with the full PBEsol correlation

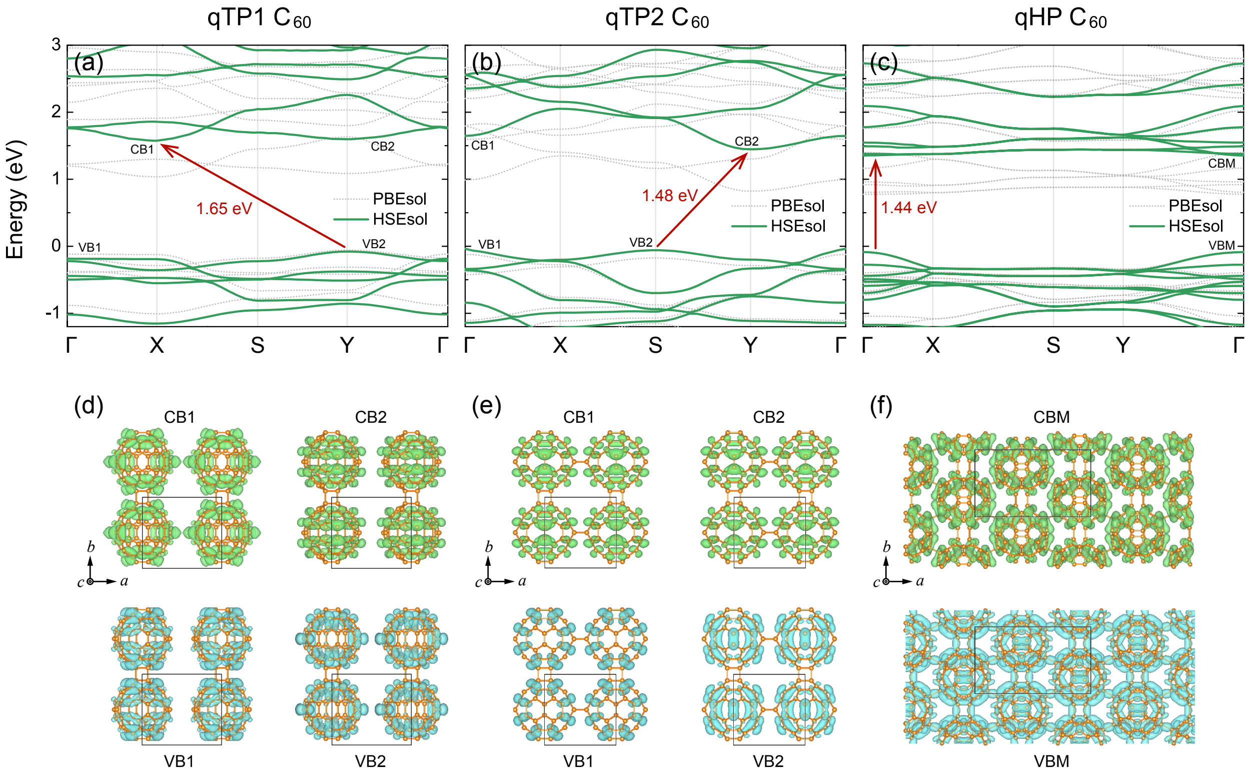

FIG. S4. Electronic structures of (a) qTP1, (b) qTP2, and (c) qHP C_{60} calculated with PBEsol functional and HSEsol hybrid functional, as well as their corresponding PBEsol partial charge density of the top valence states and the lowest conduction states in (d)-(f).

energy [16–18]. With the screening parameter decreases to 0 Å^{-1}, i.e. the long-range Hartree-Fock exchange is unscreened, the band gap further increases to 2.31 eV, as listed in Table S3.

TABLE S3. Calculated band gaps (eV) of qTP1, qTP2 and qHP C_{60} using PBEsol, HSEsol and unscreened hybrid functional PBEsol0 with their corresponding screening parameter μ (Å^{-1}).

	PBEsol	HSEsol	PBEsol0
μ	∞	0.2	0
qTP1	1.09	1.65	2.31
qTP2	0.94	1.48	2.18
qHP	0.86	1.44	2.12

For qTP2 C_{60}, the PBEsol functional predicts an indirect band gap of 0.94 eV with the VBM at Γ and the CBM at Y, while the HSEsol hybrid functional yields an indirect gap of 1.48 eV with the VBM at S and the CBM at Y. As shown in Fig. S4(b), the band structures of qTP2 C_{60} show distinct difference from qTP1 C_{60}, despite that their lattice parameters are similar.

Figure S4(c) depicts the PBEsol and HSEsol band structures of monolayer qHP C_{60}.

Monolayer qHP C_{60} possesses a direct band gap at Γ. The PBEsol band gap of 0.86 eV is in line with previous calculations[19]. The HSEsol band gap of 1.44 eV is much narrower compared to the PBEsol0 gap of 2.12 eV, which can be attributed to an increase in the dielectric screening of HSEsol[20].

G. Band alignment calculated with PBEsol, HSEsol and PBEsol0

Figure S5 summarizes the PBEsol0 band alignment of all three C_{60} monolayers, with the PBEsol and HSEsol band alignment plotted as well for comparison. In monolayer qTP1 C_{60}, the PBEsol0 CBM is 0.54 eV higher than the reduction reaction potential of H_2/H^+, which is suitable for water reduction. Moreover, the VBM is 0.54 eV lower than the oxidation potential of O_2/H_2O, which is suitable for water oxidation. Similarly, the CBM of qTP2 C_{60} is 0.50 eV higher than the reduction potential and the VBM is 0.45 eV lower than the oxidation potential. Regarding the monolayer qHP C_{60}, the CBM lies 0.46 eV above the reduction potential and the VBM is 0.43 eV below the oxidation potential.

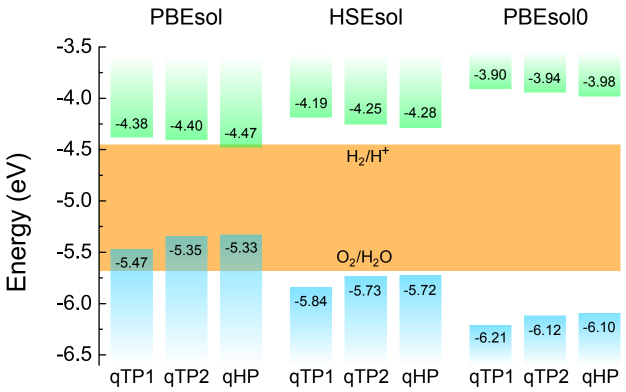

FIG. S5. Band alignment of qTP1, qTP2 and qHP C_{60} monolayers calculated with PBEsol, HSEsol and PBEsol0.

The strain effects are investigated by computing the PBEsol band alignment for all three phases with positive and negative uniaxial strains of 0.5%. In most cases the band edge shift is smaller than 0.01 eV. The maximum band edge shift in qTP1 C_{60} is -0.017 eV for CBM under compressed strain of -0.5% along b. For qTP2 C_{60}, the maximum band shift is -0.015 eV for CBM under compressed strain of -0.5% along a. Regarding qHP C_{60}, the maximum band shift is -0.029 eV for CBM under positive strain of 0.5% along a.

H. Half-reaction of water oxidation in qHP C_{60}

A supercell of 1×2 is used for qHP C_{60} with an electronic **k**-point grid of 3×3. Both the lattice constants and internal atomic coordination are fully relaxed for all the atoms. The thermal corrections at room temperature, including zero-point energy, entropy and internal thermal energy, are calculated using VASPKIT[21]. The vibrational frequencies are computed for both the adsorbed hydrogen (oxygen) atoms and the neighboring carbon atoms within a radius of 2.5 (3.0) Å. As shown in Fig. S6, at pH = 4 upon photoexcitation, all the steps are downhill, making qHP C_{60} an ideal photocatalyst for oxygen evolution reaction.

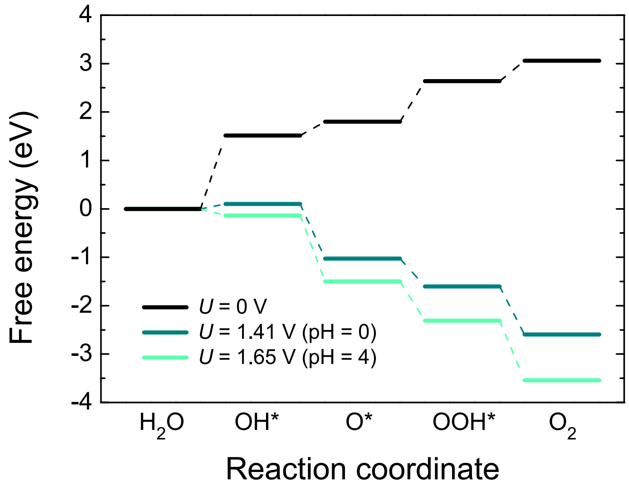

FIG. S6. Free energy diagrams of water oxidation half-reaction in qHP C_{60}.

I. Type-II band alignment of qTP2/SnTe and qTP2/PbTe heterostructures

The crystal structures of qTP2/MTe (M = Sn, Pb) heterostructures are shown in Fig. S7(a). For structural relaxation, a plane-wave cutoff of 800 eV is used with a **k**-mesh of 5×5. The zero damping DFT-D3 method of Grimme is included to describe the van der Waals interactions[1]. From the obtained crystal structures, the band structures are calculated using the PBEsol functional[11] (note that the band gap will be much larger when using hybrid functional). As shown in Fig. S7(b) and (c), both qTP2/SnTe and qTP2/PbTe heterostructures exhibit type-II band alignment, with the CBM and VBM of MTe higher than those of qTP2 C_{60}. Such alignment can separate electrons and holes efficiently.

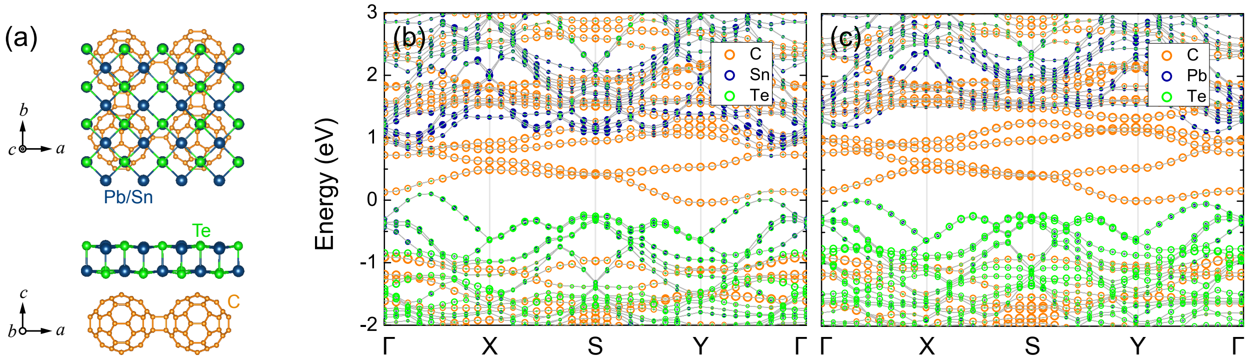

FIG. S7. (a) Crystal structures of qTP2/MTe (M = Sn, Pb) heterostructures. Electronic structures of (b) qTP2/SnTe and (c) qTP2/PbTe heterostructures calculated from PBEsol.

---

* bp432@cam.ac.uk

[1] S. Grimme, J. Antony, S. Ehrlich, and H. Krieg, J. Chem. Phys. **132**, 154104 (2010).

[2] L. Hou, X. Cui, B. Guan, S. Wang, R. Li, Y. Liu, D. Zhu, and J. Zheng, Nature **606**, 507 (2022).

[3] S. Baroni, S. de Gironcoli, A. Dal Corso, and P. Giannozzi, Rev. Mod. Phys. **73**, 515 (2001).

[4] X. Gonze, Phys. Rev. A **52**, 1086 (1995).

[5] X. Gonze, Phys. Rev. A **52**, 1096 (1995).

[6] A. Togo, F. Oba, and I. Tanaka, Phys. Rev. B **78**, 134106 (2008).

[7] A. Togo and I. Tanaka, Scripta Materialia **108**, 1 (2015).

[8] B. Peng, K. Xu, H. Zhang, Z. Ning, H. Shao, G. Ni, J. Li, Y. Zhu, H. Zhu, and C. M. Soukoulis, Adv. Theory Simul. **1**, 1700005 (2018).

[9] G. F. Lange, A. Bouhon, B. Monserrat, and R.-J. Slager, Phys. Rev. B **105**, 064301 (2022).

[10] M. Gajdoš, K. Hummer, G. Kresse, J. Furthmüller, and F. Bechstedt, Phys. Rev. B **73**, 045112 (2006).

[11] J. P. Perdew, A. Ruzsinszky, G. I. Csonka, O. A. Vydrov, G. E. Scuseria, L. A. Constantin, X. Zhou, and K. Burke, Phys. Rev. Lett. **100**, 136406 (2008).

[12] L. Schimka, J. Harl, and G. Kresse, J. Chem. Phys. **134**, 024116 (2011).

[13] B. Peng, H. Zhang, H. Shao, K. Xu, G. Ni, L. Wu, J. Li, H. Lu, Q. Jin, and H. Zhu, ACS Photonics **5**, 4081 (2018).

[14] Z. Li, B. Peng, M.-L. Lin, Y.-C. Leng, B. Zhang, C. Pang, P.-H. Tan, B. Monserrat, and F. Chen, npj 2D Materials and Applications **5**, 87 (2021).

[15] G. Su, A. Gao, B. Peng, J. Hu, Y. Zhang, F. Liu, H. Zhang, P. Zhan, and W. Wu, Nanophotonics **11**, 3149 (2022).

[16] J. P. Perdew, M. Ernzerhof, and K. Burke, J. Chem. Phys. **105**, 9982 (1996).

[17] C. Adamo and V. Barone, J. Chem. Phys. **110**, 6158 (1999).

[18] M. Ernzerhof and G. E. Scuseria, J. Chem. Phys. **110**, 5029 (1999).

[19] R. M. Tromer, L. A. Ribeiro, and D. S. Galvão, Chemical Physics Letters **804**, 139925 (2022).

[20] C. N. Savory, R. G. Palgrave, H. Bronstein, and D. O. Scanlon, Sci. Rep. **6**, 20626 (2016).

[21] V. Wang, N. Xu, J.-C. Liu, G. Tang, and W.-T. Geng, Computer Physics Communications **267**, 108033 (2021).
